# Effect of a 9‐week exercise training regimen on expression of developmental genes related to growth‐dependent fat expansion in juvenile rats

**DOI:** 10.14814/phy2.13880

**Published:** 2018-10-04

**Authors:** Hisashi Kato, Takuya Shibahara, Nazibur Rahman, Hisashi Takakura, Yoshinobu Ohira, Tetsuya Izawa

**Affiliations:** ^1^ Faculty of Health and Sports Science Doshisha University Kyotanabe City Kyoto Japan; ^2^ Graduate School of Health and Sports Science Doshisha University Kyotanabe City Kyoto Japan; ^3^ Department of Biochemistry and Molecular Biology Faculty of Biological Sciences Jahangirnagar University Savar Dhaka Bangladesh

**Keywords:** Adipogenic gene, developmental gene, exercise training, Hox gene, lipolytic gene, white adipose tissue

## Abstract

This study examined the association between changes in mRNA expression of development‐related genes including those of the homeobox (Hox) family and growth‐dependent increases in inguinal, mesenteric, and epididymal white adipose tissue (WAT) at 4, 6, 10, and 14 weeks of age in rats. We also examined the effects of a 9‐week exercise training regimen starting at 5 weeks of age on the mRNA levels of the genes of interest. *HoxC8*,* HoxC9*,* Gpc4*,* Bmpr1a*,* Pparγ*,* Pgc1α*,* Adrb3*,* Hsl*,* leptin*, and *adiponectin* in each type of WAT – except *HoxA5*,* Gpc4*, and *Pgc1α* in epididymal – showed a positive association between WAT weights and WAT mRNA levels; however, the slope of the regression lines exhibited fat depot‐specific differences. *HoxA5* showed no significant association, and *Gpc4* and *Pgc1α* showed a negative association in epididymal WAT. After exercise training, the mean *HoxA5*,* HoxC8*,* HoxC9*,* HoxC10*,* Gpc4*,* Pparγ*, and *Pgc1α* mRNA levels in inguinal WAT were outliers on the regression line between mean mRNA level and WAT weight in control rats – that is, mean *HoxA5* and *Pgc1α* mRNA level was higher, whereas *HoxC8*,* HoxC9*,* HoxC10*,* Gpc4*, and *Ppar* levels were lower in exercise‐trained rats than in same‐age controls. *Pparγγ* and adiponectin levels were upregulated in epididymal WAT, while HoxA5 was downregulated, but *HoxC9*,* Gpc4*,* Pparγ*, and adiponectin levels were upregulated in mesenteric WAT. These results suggest that some of the developmental genes tested may have fat depot‐specific roles in the growth‐dependent expansion of WAT, and that *Hox* genes that are activated in response to exercise training also vary among different WAT types.

## Introduction

White adipose tissue (WAT) in mammals is largely compartmentalized into two types – visceral adipose tissue (VAT) and subcutaneous adipose tissue (SAT) – that differ in their biological features (Gesta et al. 2007; Wronska and Kmiec 2012; Tchkonia et al. 2013; Hilton et al. 2015). For example, hormone‐stimulated adipocyte lipolysis is greater in the former than in the latter, while the opposite is true for the production and secretion of the adipokine, leptin. Furthermore, preadipocytes in abdominal SAT have a greater capacity for adipogenesis than those in VAT, and excessive accumulation of VAT is associated with increases in insulin resistance and risk of type 2 diabetes and cardiovascular disease, whereas SAT reduces type 2 diabetes risk (Wang et al. 2005; Van Gaal et al. 2006; Zhang et al. 2008). Thus, differences in regional fat distribution are thought to be an important independent risk factor for metabolic disease (Van Gaal et al. 2006).

Accumulating evidence suggests that these regional differences in the functional characteristics of different WAT types are determined not by anatomical location but by intrinsic factors including homeobox (HOX) superfamily members, which are master regulators of developmental processes (Cowherd et al. 1997; Cantile et al. 2002; Vohl et al. 2004; Gesta et al. 2006, 2007; Tchkonia et al. 2007; Yamamoto et al. 2010; Procino and Cillo 2013; Hilton et al. 2015; Brune et al. 2016; Singh et al. 2016). *HoxC9* and *HoxC10* mRNA levels are significantly higher in SAT than in WAT (Brune et al. 2016), and *HoxA5* expression is positively associated with body mass index and waist‐to‐hip ratio in humans (Gesta et al. 2006). Moreover, secreted frizzled‐related protein 2, pregnancy‐associated plasma protein A‐1, transcription factor T‐box (TBX)15, and engrailed 1 are also expressed in a fat depot‐specific manner (Gesta et al. 2006; Tchkonia et al. 2007). Fat depot‐specific differences in the expression of developmental genes have also been noted in rodents: *HoxC8* and *glypican* (*Gpc*)*4* levels are higher, whereas those of *HoxC9* and *Tbx15* are lower in VAT than in SAT (Gesta et al. 2006). In obese mice, fasting markedly enhanced the expression of *HoxC9* specifically in the mesenteric WAT but reduced *HoxC8* expression in both VAT and SAT (Yamamoto et al. 2010). Thus, although the differences are evident in the manner of expression between humans and rodents, *Hox* genes are critical determinants of WAT expansion and are thus related to fat distribution and expansion, resulting in obesity (Hilton et al. 2015; Brune et al. 2016).

WAT expansion also occurs during growth in mammals; it is possible that this is accompanied by fat depot‐specific changes in the mRNA expression of developmental genes. Furthermore, although it is well known that exercise training inhibits growth‐dependent increases in WAT weight (Izawa et al. 1985; Nomura et al. 2002; Ogasawara et al. 2012), there is little evidence that it can alter *Hox* gene expression at its endpoint. To clarify these issues, we examined development‐ and adipocyte function‐related gene expression profiles of rats during postweaning growth. We also examined chronic exercise training‐induced changes in target gene expression as the final outcome. For the developmental genes, we focused on *HoxA5*,* HoxC8*,* HoxC9*,* HoxC10*,* Tbx15*,* Short stature homeobox* (*Shox2*), *Gpc4*, and *bone morphogenetic protein receptor* (*Bmpr*)*1a* as they are well known to exhibit depot‐specific differences in their expression in rodents (Gesta et al. 2006, 2007; Yamamoto et al. 2010; Procino and Cillo 2013; Hilton et al. 2015). Furthermore, to highlight the effect of chronic exercise training, the mRNA levels of some molecules, whose functions are well known, were also determined: *adrenoceptor β3* (*Arb3*), *hormone‐sensitive lipase* (*Hsl*), *peroxisome proliferator‐activated receptor* (*Ppar*)*γ*,* pparγ coactivator* (*Pgc*)*1α*,* leptin*, and *adiponectin*.

## Materials and Methods

### Animals

Male Wistar rats (Japan SLC, Hamamatsu, Japan) with an initial body weight of approximately 100 g (4 weeks old) were housed two or three to a cage in a temperature‐controlled room at 23°C on a 12:12‐h light‐dark cycle, with free access to the standard MF pellet diet (Oriental Yeast, Japan) and water. Oriental MF contained the following ingredients per 100 g: 7.9 g moisture; 23.1 g crude protein; 4.9 g crude fat; 5.8 g crude ash; 2.8 g crude fiber; and 55.3 g N‐free extract. The animals were randomly divided into sedentary control (*n* = 20) and exercise‐trained (*n* = 5) groups. To divide the period of growth into four stages, the sedentary controls were sacrificed at 4, 6, 10, and 14 weeks of age (*n* = 5 for each time point), corresponding to postweaning, puberty, adolescence, and mature, respectively (Sengupta 2013), whereas the rats of the exercise‐trained group were subjected to exercise on a treadmill set at a 5° incline for 5 days each week for a total of 9 weeks starting at 5 weeks old according to our previously described protocol (Izawa et al. 1985; Oh‐ishi et al. 1997; Nomura et al. 2002; Ogasawara et al. 2012). Briefly, the initial training intensity was 15 m/min for 20 min; thereafter, the running speed and duration were progressively increased until 6 weeks, when the rats were running continuously at 30 m/min for 90 min. Control rats were not required to run on a treadmill. Exercise‐trained rats were sacrificed at least 36 h after the last exercise session. Rats were anesthetized by intraperitoneal injection of sodium pentobarbital (5 mg/100 g body weight; Abbott Laboratories, Lake Bluff, IL, USA), and epididymal, mesenteric, and inguinal WATs were rapidly removed. All experiments in this study were approved by the Animal Care Committee of Doshisha University.

### Real‐time (RT)‐PCR

Total RNA was prepared from each WAT sample using ISOGEN (Nippon Gene, Tokyo, Japan). RNA samples were diluted in diethyl pyrocarbonate water and stored at −80°C until RT‐PCR analysis. The sequences of oligonucleotide primers and probes specific for the various targets are shown in Table 1. For synthesis of first‐strand cDNA, 1 *μ*g of RNA was added to RNase‐free water along with oligo(dT) and RNase inhibitor (Boehringer Mannheim, Mannheim, Germany) in a final volume of 50 *μ*L. The reaction mixture was incubated at 70°C for 10 min. First‐strand synthesis buffer (Gibco, Grand Island, NY, USA), 100 mmol/L dithiothreitol, and dNTP were added to the tube followed by incubation at 37°C for 2 min. Superscript II reverse transcriptase (Gibco) was then added to the tube and the reaction was carried out in a Chromo4 PCR system (Bio‐Rad, Hercules, CA, USA). SYBR Green Supermix (Bio‐Rad) was used in a final reaction volume of 20 *μ*L with 20 ng of each primer and 20 ng of cDNA template. Cycling conditions were as follows: initial denaturation at 95°C for 5 min, followed by 40 cycles of annealing for 1 min at the temperature indicated for each primer, and extension at 72°C for 30 sec. The temperature transition was 3°C/sec for all reactions. Samples containing the largest amount of each target were used to generate standard curves (1‐, 2‐, 10‐, 20‐, and 100‐fold dilutions of transcribed cDNA). All standard curves were linear, with *r* ^2^ values >0.98. In the same run, cycle threshold values of samples (20‐fold diluted cDNA) were used to determine relative expression levels. The expression of 18S rRNA was measured as an internal control in the same manner; the level was relatively stable across samples, with differences consistently <20%. Target mRNA levels were normalized to that of 18S rRNA in each sample. PCR reactions were run in triplicate.

**Table 1:** Sequences of primers used in this study

Gene	Sense primer (5′→3′)	Antisense primer (5′→3′)
*HoxA5*	AGAGGTCATCAGGCAGGATTTAC	GCGGTCGTTTGTGCGTCTAT
*HoxC8*	TGCAATATCCCGACTGTAAATCCTC	CCAAGGTCTGATACCGGCTGTAA
*HoxC9*	CGGCAGCAAGCACAAAGAG	ACCGACGGTCCCTAGTTAAATACA
*HoxC10*	CCAGACACCTCGGATAAT	TCTCCAATTCCAGCGTCT
*Tbx15*	GCCTGGATCCACATCAGCAATAC	GCCACCATCCACTTGGAGCTA
*Shox2*	ACTATCCAGACGATTTCATGCG	TTCGATTTTGAAACCAAACCTGATC
*Gpc4*	GGAGACCTTGATTTAGAGTTGGAACA	CACATCGATGGGATCCATAAC
*Bmpr1a*	GATGCTGCCTGGTTGATGATG	TGGCCACAAATACCGTCCTG
*Adrb*	AGCTAGCCCTGTTGCGTCCA	GGAGAGTTGCGGTTCCTGGG
*Hsl*	CCTACTACACAAATCCC	CTCAAAGAAGAGCACTC
*Pparγ*	GGAGCCTAAGTTTGAGTTTGCTGTG	TGCAGCAGGTTGTCTTGGATG
*Pgc1a*	GCACTGACAGATGGAGACGTGAC	TCATTGTAGCTGAGCTGAGTGTTGG
*Leptin*	TGACAAACAGGTTACAFFACCAGA	ACGAAACCCGATCCAGTTCA
*Adiponectin*	GGGAGACGCAGGTGTTCTTG	CGCTGAATGCTGAGTGATACATG
*Rps18*	AAGTTTCAGCACATCCTGCGAGTA	TTGGTGAGGTCAATGTCTGCTTTC

*Adrb3*, adrenoceptor *β*_3_; *Bmpr1a*, bone morphogenetic protein receptor type 1a; *Gpc4*, glypican 4; *HoxA5/C8/C9/C10*, homeobox A5/C8/C8/C9/C10; *Hsl*, hormone‐sensitive lipase; *Pparγ*, peroxisome proliferator‐activated receptor *γ*;* Pgc1α*, PPAR*γ* coactivator 1*α*;* Rps18*, ribosomal protein S1a/S18; *Shox2*, short stature homeobox 2; *Tbx15*, T‐box transcription factor 15.

### Statistical analysis

Values represent the mean ± SE. Fat depot‐specific differences at each age in weeks and after exercise training (with the mRNA level of inguinal WAT at each stage set to 1) were evaluated by one‐way ANOVA followed by Bonferroni's test. Results were considered significant at *P* < 0.05. Simple linear regression analysis between the fold change of mRNA expression and that of WAT weight in control rats (with the mRNA level and WAT weight of 4‐week‐old rats set to 1) was carried out, and the linear regression lines were compared by analysis of covariance. We tested whether the mean value of parameters from exercise‐trained rats was an outlier on the regression line between the mean rate of increase in mRNA expression and WAT growth rate in control rats. Outlier diagnostics were determined by leverage. Results were considered significant at *P* < 0.05.

## Results

### Changes in body and WAT weights

Body weight and mass of inguinal, epididymal, and mesenteric WAT increased as a function of growth in control rats (Table 2). The final mean body weight and the absolute mass as well as relative weight (% body weight) of the three types of WAT were significantly lower in exercise‐trained than in control rats. The inhibitory effect of exercise training on growth‐dependent increases in body weight was significant after the sixth week of the exercise training regimen (results not shown).

**Table 2:** Body weight in rats (g) and mass of various types of white adipose tissue (mg/g body weight)

		Body weight (g)	Adipose tissue weight		
			Inguinal	Epididymal	Mesenteric
4 weeks old	(g)	73.8 ± 1.6	558 ± 11	288 ± 19	362 ± 25
	(mg/g body weight)		7.56 ± 0.08	3.89 ± 0.22	4.89 ± 0.26
6 weeks old	(g)	146.4 ± 2.0*	1368 ± 82*	1082 ± 86*	748 ± 55*
	(mg/g body weight)		9.34 ± 0.52*	7.38 ± 0.55*	5.10 ± 0.35*
10 weeks old	(g)	277.6 ± 4.4†	2806 ± 120†	3522 ± 183†	1830 ± 95†
	(mg/g body weight)		10.10 ± 0.38†	12.72 ± 0.76†	6.60 ± 0.34†
14 week old	(g)	328.8 ± 4.9!	5188 ± 284!	6816 ± 322!	3648 ± 264!
	(mg/g body weight)		15.83 ± 1.04!	20.73 ± 0.93!	11.06 ± 0.68!
Exercise‐trained (14 weeks old)	(g)	270.4 ± 5.1§	3516 ± 155§	4636 ± 269§	2216 ± 97§
	(mg/g body weight)		12.99 ± 0.41§	17.17 ± 1.02§	8.20 ± 0.34§

Values represent mean ± SE for 5 rats in each group. *P ≤ 0.05 versus 4 weeks old, ^†^ *P* ≤ 0.05 versus 6 weeks old, ^!^ *P* ≤ 0.05 versus 10 weeks old, ^§^ *P* ≤ 0.05 versus 14 weeks old.

### Fat depot‐specific differences in mRNA expression of developmental and other genes

**Figure 1:**
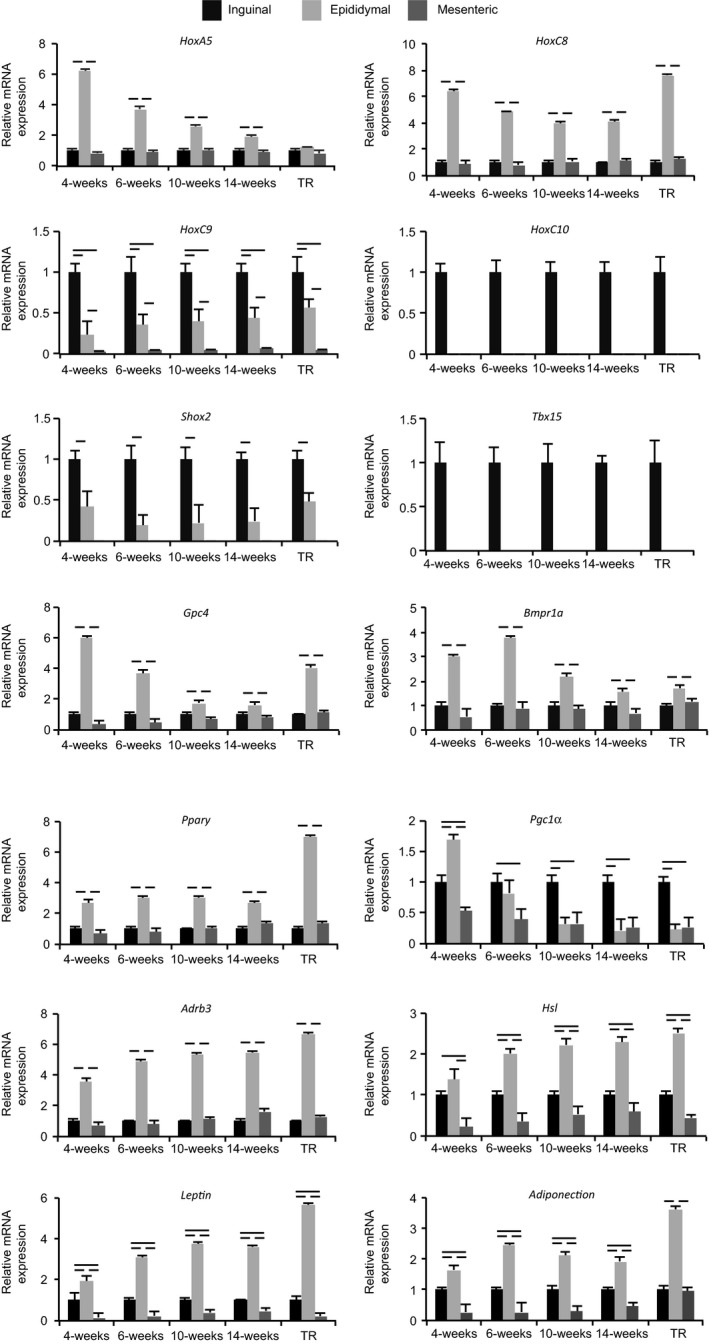
Expression of genes related to development and adipose tissue functions in inguinal, epididymal, and mesenteric WAT from control and exercise‐trained rats (TR). Expression levels of *HoxA5*,* HoxC9*,* HoxC10*,* Tbx15*,* Shox2*,* Gpc4*, and *Bmpr1a* as well as *Pparγ*,* Pgc1α*,* Adrb3*, and *Hsl*, were quantified by real‐time PCR. Five independent samples, each from a different rat (*n* = 5), were each analyzed in duplicate. Levels were normalized to those of 18S rRNA for each sample. Values in bar graphs represent relative density of inguinal adipose tissue at each age (in weeks) or in TR rat (set to 1.0), and are shown as mean ± SE. The bars indicate significant difference (*P* < 0.05 or less) between depots.

We evaluated fat depot‐specific differences in mRNA levels in each type of WAT from control and exercise‐trained rats. To this end, as shown in Figure 1, the values for epididymal and mesenteric WAT were related to the values for inguinal WAT (set to 1) at each age in weeks and after exercise training. Comparison of the relative expression of the genes tested at 14 weeks of age in rats is summarized in Table 3. The mRNA expression levels of *HoxA5*,* Gpc4*,* Bmpr1a*,* Pparγ*, and *Arb3* were more highly expressed in epididymal as compared to other types of WAT. *Tbx15* mRNA was detected in inguinal WAT only, and *HoxC9* mRNA was more highly expressed in inguinal as compared to other WATs. *Shox2* mRNA was expressed in inguinal and epididymal but not in mesenteric WAT, and the level was higher in inguinal than in epididymal WAT. *HoxC10* mRNA was expressed in inguinal WAT but almost undetectable in the other WAT types. *Leptin* and *adiponectin* mRNAs were most highly expressed in epididymal as compared to other types of WAT and were higher in inguinal WAT than in mesenteric WAT.

**Table 3:** Comparison of the expression levels of the genes in three WATs from sedentary control rats

*Genes*	Expression		
	Inguinal	Epididymal	Mesenteric
*HoxA5*	**+**	**++**	**+**
*HoxC8*	**+**	**++**	**+**
*HoxC9*	**+++**	**++**	**+**
*HoxC10*	**+**	Trace	Trace
*Tbx15*	**+**	ND	ND
*Shox2*	**++**	**+**	ND
*Gpc4*	**+**	**++**	**+**
*Bmpr1a*	**+**	**++**	**+**
*Adrb*	**+**	**++**	**+**
*Hsl*	**++**	**+++**	**+**
*Pparγ*	**+**	**++**	**+**
*Pgc1α*	**++**	**+**	**+**
*Leptin*	**++**	**+++**	**+**
*Adiponectin*	**++**	**+++**	**+**

Original data are shown in Figure 1. Ing, inguinal WAT; Epi, epididymal WAT; Mes, mesenteric WAT; ND, no mRNA detected; Trace, trace mRNA detected. Plus sign (+) represents the relative degree of the expression of the genes.

These fat depot‐specific differences in mRNA expression were observed at each early postnatal time point examined. Meanwhile, *Pgc1α* was most highly expressed in epididymal WAT at 4 weeks, but was gradually downregulated in epididymal WAT, and was most highly expressed in inguinal WAT after 10 weeks of age. *Hsl* mRNA was most highly expressed in epididymal WAT from 6 to 14 weeks of age, but the level did not differ significantly between inguinal and epididymal WAT at 4 weeks of age. Finally, with the exception of *HoxA5* and *Hsl*, fat depot‐specific differences in mRNA expression in the exercise‐trained rats were similar to those observed in control rats of the same age.

### Correlation between rate of increase in mRNA expression and fat pad growth rate in sedentary control rats

**Figure 2:**
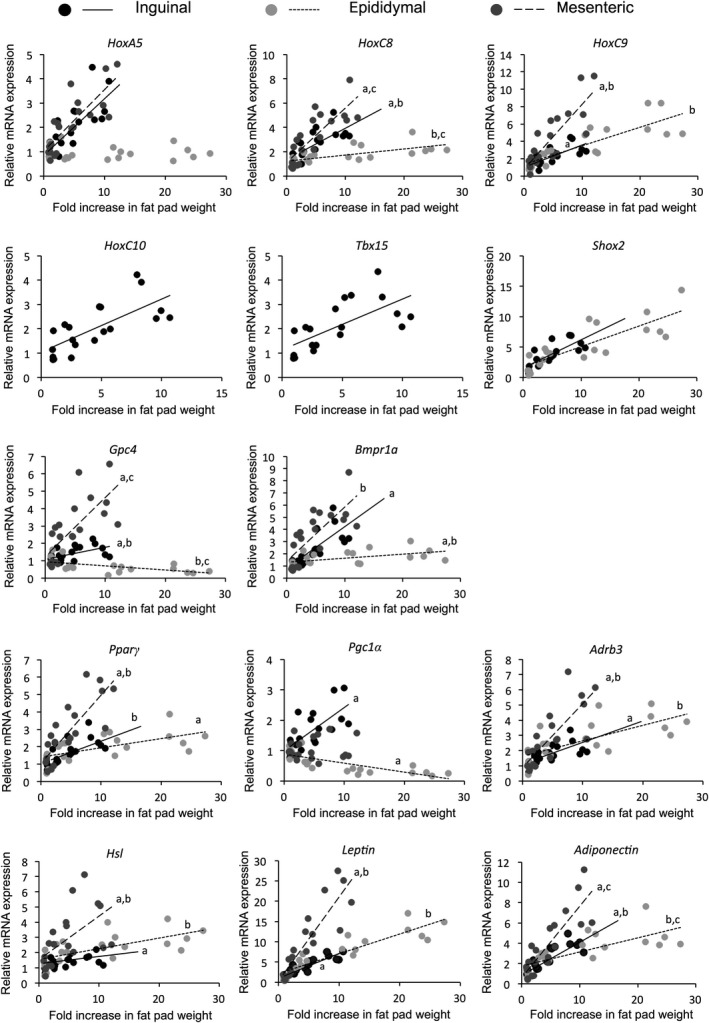
Correlation between the rate of increase in fat pad weight with the mRNA expression of genes in control rats (*n* = 5 at each time point). Levels were normalized to those of 18S rRNA for each sample. The value of each dot is the optical density of each adipose tissue sample from 4‐week‐old rats (set to 1.0). The association was calculated by simple linear regression analysis, and linear regression lines were compared by analysis of covariance. Lines with the same superscript had significantly different slopes (*P* < 0.05).

We next examined whether the observed changes in the gene expression were associated with growth‐dependent increases in WAT weight in sedentary control rats. For most of the examined genes, there was a significant positive correlation between the rate of increase in mRNA expression and WAT growth rate (Fig. 2); the coefficients of determination (R^2^) and relative comparison of the slope of the regression lines in three WATs are summarized in Table 4. As shown in Figure 2 and Table 4, most genes expressed in WAT showed a positive correlation between increases in mRNA level and WAT weight. However, the slope of the regression line showing the association of the rate of increase in *HoxC8*,* Gpc4*, and *adiponectin* mRNA level with WAT growth rate differed significantly among the three types of WAT. The slope for *HoxC8* mRNA level and that for *adiponectin* were most steep in mesenteric WAT and less steep in epididymal than in other WATs, although the mRNA expression levels of these transcripts were most highly in epididymal WAT (Fig. 1 and Table 3). Furthermore, a negative slope was observed for *Gpc4* mRNA level in epididymal WAT. *HoxC9* mRNA was most highly expressed in inguinal WAT (Fig. 1 and Table 3), but the slope of the regression line between *HoxC9* mRNA level and WAT weight was steeper in mesenteric than in other WATs. The slope of the regression line between the rate of increase in *Bmpr1a* mRNA level and WAT growth rate was steeper in inguinal and mesenteric than in epididymal WAT, but *Bmpr1a* mRNA was most highly expressed in epididymal WAT (Fig. 1 and Table 3). Furthermore, there was no significant correlation in *HoxA5* mRNA level in epididymal WAT. The slope of the regression line between the rate of increase in *Shox2* mRNA expression and WAT growth rate was the same between inguinal and epididymal WAT, but it was highly expressed in inguinal compared with epididymal WAT (Fig. 1 and Table 3).

**Table 4:** Coefficient of determination (*R* ^2^) between rate of increase in mRNA expression and fat pad growth rate, and relative comparison of the slope of the regression lines in three WATs from sedentary control rats

*Genes*	*R* ^2^			Slope		
	Ing	Epi	Mes	Ing	Epi	Mes
*HoxA5*	0.623†	0.005	0.654†	**+**		**+**
*HoxC8*	0.557†	0.419†	0.656†	**++**	**+**	**+++**
*HoxC9*	0.558†	0.693†	0.710†	**+**	**+**	**++**
*HoxC10*	0.518†	Trace	Trace	**+**		
*Tbx15*	0.468†	ND	ND	**+**		
*Shox2*	0.634†	0.686†	ND	**+**	**+**	
*Gpc4*	0.294*	−0.321†	0.522†	**+**		**++**
*Bmpr1a*	0.618†	0.255*	0.574†	**++**	**+**	**++**
*Adrb*	0.450†	0.491†	0.638†	**+**	**+**	**++**
*Hsl*	0.297*	0.415†	0.327†	**+**	**+**	**++**
*Pparγ*	0.445†	0.374†	0.664†	**+**	**+**	**++**
*Pgc1α*	0.512†	−0.578†	0.015	**+**		
*Leptin*	0.814†	0.820†	0.797†	**+**	**+**	**++**
*Adiponectin*	0.747†	0.541†	0.734†	**+**	**+**	**++**

Original data are shown in Figure 2. Ing, inguinal WAT; Epi, epididymal WAT; Mes, mesenteric WAT; ND, no mRNA detected; Trace, trace mRNA detected. Plus sign (+) represents the relative degree of the steepness of the angle (slope) of the regression line. *P ≤ 0.05, ^†^ *P* ≤ 0.01.

The slope of the regression line showing the association of the rate of increase in *Adrb3*,* Hsl*,* Pparγ*, and *leptin* with WAT growth rate was also steeper in mesenteric than in the other WATs, but the mRNA expression levels of these transcripts were most highly expressed in epididymal WAT (Fig. 1 and Table 3). The rate of increase in *Pgc1α* mRNA was positively correlated with the rate of growth of inguinal WAT; there was a negative correlation in epididymal WAT and no correlation in mesenteric WAT.

### Effects of exercise training on mRNA expression levels

In the present study, growth‐dependent increase in body weight was significantly inhibited after the sixth week of exercise training regimen (results not shown). Protocols similar to the exercise training regimen used in this study have been shown to enhance epididymal adipocyte lipolysis (Izawa et al. 1985; Nomura et al. 2002; Ogasawara et al. 2012) and skeletal muscle citrate‐synthase activity (Oh‐ishi et al. 1997) 9 weeks after exercise training regimen. On the other hand, the significant inhibition of growth‐dependent increase in the weight of several adipose tissues (epididymal, perirenal, and omental WAT) and the enhanced hormone‐stimulated lipolysis in epididymal adipocytes were found no earlier than the sixth week of the same protocol of exercise training regimen (our unpublished observations). Thus, the adaptation of adipose tissue and respiratory enzymes in skeletal muscles to chronic exercise training should be evidently found 9 weeks after exercise training regimen. To verify the effects of chronic exercise training on the expression of parameters during the growth of rats, we examined changes in the parameters tested as the endpoint of adaptation to chronic exercise training in the final outcome.

**Figure 3:**
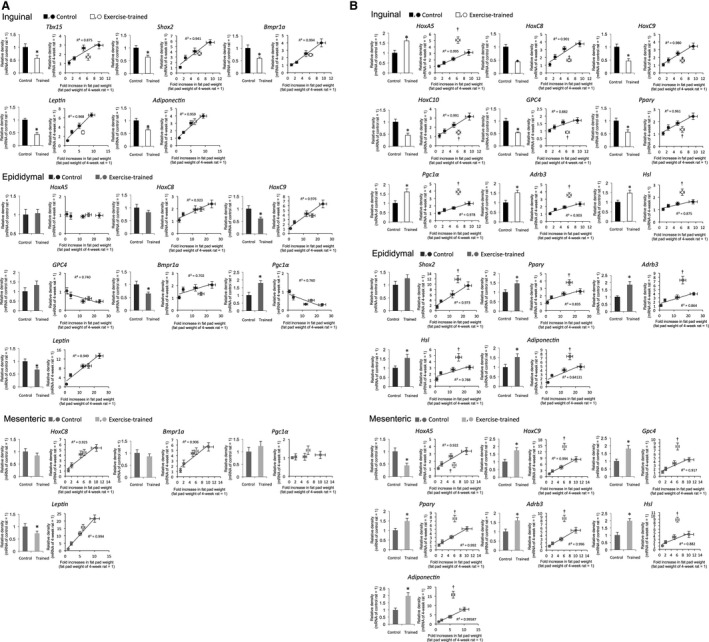
Comparison of mRNA expression levels of genes between exercise‐trained (*n* = 5) and age‐matched (14 weeks old) control rats (*n* = 5) (bar graphs), and correlation between rate of increase in fat pad weight and mRNA expression of genes in control (*n* = 5 at each time point) and exercise‐trained (*n* = 5 at 14 weeks age) rats (correlation diagrams). Levels were normalized to those of 18S rRNA for each sample. Values in the bar graphs (the mean ± SE) are related to the relative density of each adipose tissue sample from 14‐week‐old control rats (set to 1.0). In the correlation diagram, each dot represents the relative density of adipose tissue from 4‐week‐old control rats (set to 1.0), and is shown as the mean value ± SE. The mean values of control rats were calculated from the data in Figure 2. The association was calculated by simple linear regression analysis between the mean value of the rate of increase in mRNA expression and growth rate of WAT in control rats (either mRNA or body weight of 4‐week‐old rat set to 1). The significance of differences between control and exercise‐trained rats at 14 weeks old in the bar graphs was assessed by analysis of variance with Bonferroni's post hoc test. *P < 0.05 vs. 14 weeks old. When the mean value of exercise‐trained rats was an outlier in the regression line between the mean value of rate of increase in mRNA expression and that of the growth rate of WAT in control rats, outlier diagnostics were determined by leverage. The graphs indicating non‐outlier are arranged in A, and those indicating outlier in B. ^†^ *P* < 0.05. All coefficients of determination (*R* ^2^) between fat pad weight and genes related to development and adipocyte function in sedentary control rats were statistically significant (*P* < 0.05).

Exercise training altered the expression levels of most of the genes compared to respective controls (Fig. 3). However, since exercise training inhibited the growth‐dependent increase in WAT weight (Table 2) and the mRNA level of most tested genes was dependent on the increase in WAT weight (Fig. 2), we verified whether the observed changes in mRNA levels were due to the lower WAT in exercise‐trained rats relative to controls of the same age. We determined outlier diagnostics by leverage: the mean mRNA expression levels of genes in exercise‐trained rats were plotted on the regression line between mean values of the rate of increase in mRNA expression and those of WAT growth rate in control rats. In this analysis, given that the observed changes in mRNA levels after exercise training were associated with changes in WAT weight, we predicted that the mRNA expression level of these genes in exercise‐trained rats would be located on a regression line between the mRNA level of the same gene and WAT weight in control rats. On the other hand, when the mRNA expression of a given gene in exercise‐trained rats was a statistical outlier from the regression line in control rats, its change could be due to the effect of exercise training per se. The results showed that some of the developmental genes were dependent on WAT expansion in a fat depot‐specific manner: mRNA levels of *Tbx15*,* Shox2*,* Bmpr1a*,* leptin*, and *adiponectin* were specifically associated with the expansion of inguinal WAT; *HoxC8*,* HoxC9*,* Bmpr1a*, and *leptin* levels were positively associated, whereas those of *Gpc4* and *pgc1α* were negatively associated with the expansion of WAT; and *HoxC8*,* Bmpr1a*, and leptin levels were associated with the expansion of mesenteric WAT (Fig. 3A).

On the other hand, the mRNA levels of some *Hox* genes in exercise‐trained rats were outliers on the regression line between the rate of increase in mRNA expression and WAT growth rate in control rats (Fig. 3B). In inguinal WAT, exercise training enhanced the expression of *HoxA5* but reduced that of *HoxC8*,* HoxC9*,* HoxC10*, and *Gpc4*. In epididymal WAT, none of the developmental genes were altered by exercise training, whereas in mesenteric WAT, exercise training reduced the expression of *HoxA5* but enhanced that of *HoxC9* and *Gpc4*.

*Adrb3* and *Hsl* mRNA levels were increased in all three WATs after exercise training, and each was an outlier on the regression line between the rate of increase in the expression of each mRNA and WAT growth rate in control rats. *Pparγ* mRNA expression was also an outlier on this regression line, whereas *Pgc1α* expression was an outlier in inguinal WAT only. *Pparγ* mRNA expression was upregulated in epididymal and mesenteric WAT but downregulated in inguinal WAT after exercise training. *Pgc1α* expression was increased by exercise training in inguinal WAT, whereas no changes were observed in the other two WAT types in response to exercise training. *Adiponectin* mRNA level was increased in epididymal and mesenteric WAT after exercise training, and each was an outlier on the regression line between the rate of increase in the expression of each mRNA and WAT growth rate in control rats.

## Discussion

Recent studies have shown that developmental genes such as *HoxA1*,* HoxA5*,* HoxC4*,* HoxC8*,* HoxC9*,* HoxA10*,* engrailed 1*,* Gpc4*,* nuclear receptor subfamily 2 group F member 1*,* secreted frizzled‐related protein 2*,* Shox2*,* Tbx15*, and *thrombomodulin* are expressed in preadipocytes and mature adipocytes; moreover, several *Hox* genes show fat depot‐specific expression (Gesta et al. 2007; Hilton et al. 2015; Brune et al. 2016; Vohl et al. 2004; Procino and Cillo 2013; Gesta et al. 2006; Cowherd et al. 1997; Cantile et al. 2002; Singh et al. 2016; Yamamoto et al. 2010; Tchkonia et al. 2007). Our results confirm these earlier observations – that is, we observed fat depot‐specific expression of several *Hox* genes. At 14 weeks of age, *HoxC9* was most highly expressed in inguinal WAT, whereas *HoxA5*,* HoxC8*,* Gpc4*, and *Bmpr1a* showed the highest expression in epididymal WAT. On the other hand, none of the genes was more highly expressed in mesenteric as compared to the other types of WAT, and *Shox2* was not detected in this WAT type. As previously reported in rodents (Gesta et al. 2007; Yamamoto et al. 2010; Hilton et al. 2015), *Tbx15* was expressed only in inguinal WAT, whereas *HoxC10* transcript was barely detectable in epididymal and mesenteric WAT.

Fat depot‐specific differences in the expression of most *Hox* genes were observed throughout the early postnatal period in control rats. Although the precise underlying mechanism is unclear, it may be associated with fat depot‐specific characteristics inherent to preadipocytes. Distinct preadipocyte subpopulations are distributed in SAT and VAT that differ in terms of propensity for apoptosis, proliferation, and adipogenesis in humans (Tchkonia et al. 2005) and in rodents (Macotela et al. 2012). Differences in developmental gene expression profiles have also been observed between VAT and SAT preadipocytes (Gesta et al. 2006). Thus, fat depot‐specific characteristics inherent to preadipocytes are thought to contribute to regional differences in WAT function (Kirkland and Dobson 1997; Tchkonia et al. 2005). Preadipocyte numbers increase in both perirenal and epididymal fat depots during maturation (Kirkland and Dobson 1997), and it has been suggested that the proportion of preadipocytes relative to total cells derived from WAT is at most 5–8% (Tchoukalova et al. 2004). However, the adipocyte population has a rapid turnover: 4.8% of preadipocytes are replicating at any given time and between 1 and 5% are replaced daily in murine WAT (Rigamonti et al. 2011). Thus, fat depot‐specific differences in *Hox* gene expression may be maintained during the early stages of life in rats.

*Hox* genes play a role in regulating WAT expansion (Hilton et al. 2015; Seifert et al. 2015). Our regression analysis revealed that most developmental genes examined were also positively associated with the growth‐dependent expansion of WAT. In addition, we found that the role of *Hox* genes in regulating growth‐dependent WAT expansion, if any, may vary across WAT types regardless of differences in mRNA expression level in individual WATs (Tables 3 and 4). These variations seem to be related to the reported differences in the manner of expansion, which involves adipocyte hypertrophy and/or hyperplasia, between subcutaneous and visceral WAT (Maslowska et al. 1993; DiGirolamo et al. 1998; Macotela et al. 2012). DiGirolamo et al. (1998) reported that during the normal growth of Wistar rats, from 7 weeks to 15 months of age, the cumulative growth of the two intraabdominal fat depots (mesenteric and epididymal) was mostly due to hypertrophy, whereas that of the other two depots (retroperitoneal and inguinal) was predominantly due to hyperplasia. The in vitro differentiation ability of subcutaneous stromal vascular cells is also greater than that of gonadal and visceral ones in mice (Maslowska et al. 1993; Macotela et al. 2012). Thus, it is of interest to assume that the depot‐specific variations in the slope of the regression lines may be associated with the distinct differences in the manner of expansion between subcutaneous and visceral WAT.

However, it should be noted that the proposed roles of the genes in adipogenesis were not always enough to prove the relationship between the fat depot‐specific variations in the slope for the genes and the distinct differences in the manner of expansion between subcutaneous and visceral WAT. For example, since HoxA5 and BMP2 have a functional role in adipogenesis in rodents (Chen et al. 2001; Lee et al. 2014; Seifert et al. 2015; Cao et al. 2018), the finding that the slope of the regression line showing the association of either *HoxA5* or *Bmpr1a*, a receptor for BMP, with WAT growth was not different between inguinal subcutaneous and mesenteric visceral, implies that both transcripts stimulate adipogenesis to the same extent in subcutaneous and visceral WAT. However, this is inconsistent with the findings of DiGirolamo et al. (1998), which showed that adipocyte hyperplasia and hypertrophy assumed the responsibility for growth‐dependent expansion in inguinal and mesenteric WAT, respectively. The observed depot‐specific association between *Pparγ* and *Gpc4* mRNA was also inconsistent with their reported roles for adipogenesis. *Gpc4* expression was found to increase during the differentiation of stromal vascular fraction cells along with an increase in *Pparγ* expression (Singh et al. 2016), and PPAR*γ* activation increased *Gpc4* mRNA and protein levels in subcutaneous WAT, while having no effect on visceral WAT (Liu et al. 2014). In the present study, however, the association of each gene expression for growth‐dependent WAT expansion was greater in mesenteric WAT than in inguinal WAT (Fig. 2 and Table 4). Furthermore, epididymal WAT, a visceral type of WAT, exhibited a negative relationship between *Gpc4* and WAT growth, even though growth‐dependent increase in *Pparγ* mRNA expression was found. Collectively, a monistic explanation for the *Hox* network regulating WAT plasticity may be difficult. This may be because the individual *Hox* genes composed of networks may influence each other to regulate WAT expansion and because their roles may also be impacted by variations in local factors in the in vivo condition (Fried et al. 2015).

With such a limitation, our data obtained from the effects of chronic exercise training on the genes, as outcomes, display new and unique relationship between *Hox* genes and fat expansion. We examined the effect of exercise training – which reduced WAT weight relative to age‐matched sedentary control animals – on the expression of developmental genes. An analysis of outliers revealed that some developmental genes were specifically dependent on WAT expansion. That is, *Tbx15*,* Shox2*,* Bmpra1*,* leptin*, and *adiponectin* transcript levels were associated with the expansion of inguinal WAT; the same was true for *HoxC8*,* HoxC9*,* Bmpra1*, and *leptin* in epididymal WAT and for *HoxC8*,* Bmpr1a*, and *leptin* in mesenteric WAT (Fig. 3A); however, *Gpc4* and *Pgc1α* were negatively dependent on WAT expansion in epididymal WAT. *HoxA5* and *Pgc1α* were not associated with WAT expansion in epididymal and mesenteric WAT, respectively. The expression of other developmental genes corresponded to exercise training‐induced inhibition of WAT expansion – i.e., *HoxA5*,* Hoxc8*,* Hoxc9*,* Hoxc10*,* Gpc4*,* Pparγ*,* Pgc1α*,* Adrb3*, and *Hsl* in inguinal WAT; *Shox2*,* Pparγ*,* Adrb3*,* Hsl*, and adiponectin in epididymal WAT; and *HoxA5*,* Hoxc9*,* Gpc4*,* Pparγ*,* Adrb3*,* Hsl*, and adiponectin in mesenteric WAT. Thus, the largest number of genes was altered in response to exercise training in inguinal WAT, and lipolytic genes were upregulated in all three WAT types. *Pparγ* expression was also altered in response to exercise training in all three WATs; however, it was increased in epididymal and mesenteric but decreased in inguinal WAT.

The physiological significance of exercise training‐induced changes in gene expression is unclear. However, based on the reported effects of exercise training on WAT morphology and function, we speculated that some *Hox* genes modulate WAT function in response to exercise training. For instance, the observed downregulation of *HoxC8* and *HoxC10* mRNA in inguinal WAT may be associated with exercise training‐induced recruitment of beige adipocytes (Stanford et al. 2015); it has been reported that downregulation of *HoxC8* in preadipocytes led to browning of adipocytes during preadipocyte differentiation (Mori et al. 2012), while *HoxC10* is considered as a negative regulator of adipocyte browning (Ng et al. 2017). Furthermore, expression of mitochondrial biogenesis and uncoupling protein 1 – as an index of browning – is required for enhanced expression of *Pgc1α* mRNA. In this study, upregulation of *Pgc1α* mRNA was detected in inguinal WAT of exercise‐trained rats (Fig. 3B); it is thus likely that exercise training‐induced downregulation of *HoxC8* and *HoxC10* is associated with adipocyte browning in inguinal WAT. This is further supported by the observation that *Tbx15* was expressed only in inguinal WAT. *Tbx15* plays an important role in the differentiation of brown and white adipocytes in the inguinal but not in the epididymal depot (Gburcik et al. 2012). Thus, as summarized in a recent review (Stanford et al. 2015), exercise training‐induced adipocyte browning may occur to a greater extent in inguinal than in other types of WAT.

We expected that the observed upregulation of *Adrb3* and *Hsl* mRNA level after exercise training was associated with the well‐known exercise training‐induced enhancement of adipocyte lipolysis in response to hormones. However, our data on the change in *Shox2* mRNA expression contradict its presumed role of positively regulating *Adrb3* mRNA expression (Lee et al. 2016). *Shox2* mRNA expression was not detected in mesenteric WAT; furthermore, in inguinal WAT, the level of transcript decreased with weight after exercise training and did not differ relative to the control group in epididymal WAT. Nevertheless, all three types of WATs showed a marked increase in *Adrb3* mRNA expression after exercise training. It is possible that genes other than *Shox2* modulate the exercise training‐enhanced expression of *Adrb3* in WATs, particularly in mesenteric WAT.

As we have previously shown that the exercise training‐induced changes in leptin mRNA expression depended on the reduced size of adipocytes, but not on TR *per se* (Miyazaki et al. 2010), leptin transcript level was associated with the expansion of WAT. On the other hand, adiponectin mRNA levels were closely associated with inguinal WAT weight. This is inconsistent with our previous finding that exercise training amplified the adipocyte‐size‐dependent increases in adiponectin mRNA expression in both inguinal and epididymal WAT (Miyazaki et al. 2010). Even though the reason is unclear, the samples used for mRNA measurements are different between the present study and our previous study; the samples for the former were adipose tissues and those for the latter were isolated adipocytes. Data on cell size and/or number and further correlational analyses may be required to support our findings.

There were several limitations to this study. First, the number of rats (*n* = 5 per each group) may not be sufficient to adequately compare groups. However, we calculated strength of association using one‐way ANOVA for fat depot‐specific difference in each mRNA expression, which indicated that values of *η* ^2^ ranged from 0.56 to 0.94. This range means that effect size is large. Second, our analysis was exclusively based on the mRNA levels of the examined genes. In order to verify the physiological significance of our observations, information on protein expression is necessary. Third, lack of data on cellularity in this study does not allow us to explain exactly how the *Hox* genes regulate growth‐dependent fat expansion. There are likely large fluctuations in the cellularity of each adipose tissue during growth and development. Finally, the present data are limited to male rats and white adipose tissue. It is well known that there are sex differences in biological functions of adipose tissues (Fried et al. 2015) and that brown adipose tissue play a crucial role in body fat control (Hilton et al. 2015).

## Conclusion

We demonstrated that fat depot‐specific differences in *Hox* genes were maintained during the early postnatal period in rats, and that such differences were also observed in the relationship between developmental gene expression and growth‐dependent WAT expansion irrespective of variations in mRNA expression levels in individual WAT types. Additionally, we found that developmental genes were divided into two types: those dependent on exercise training‐induced changes in WAT weight and those that may be directly altered by exercise training. These results indicate that *Hox* genes play distinct roles in exercise training‐induced changes in WAT expansion during periods of growth.

## Conflict of Interest

The authors declare that they have no competing interests.
